# The core functions and forms paradigm throughout EPIS: designing and implementing an evidence-based practice with function fidelity

**DOI:** 10.3389/frhs.2023.1281690

**Published:** 2024-01-16

**Authors:** Alec Terrana, Clare Viglione, Kyung Rhee, Borsika Rabin, Job Godino, Gregory A. Aarons, Jessica Chapman, Blanca Melendrez, Margarita Holguin, Liliana Osorio, Pradeep Gidwani, Cynthia Juarez Nunez, Gary Firestein, Eric Hekler

**Affiliations:** ^1^ School of Medicine, University of California San Diego, San Diego, CA, United States; ^2^ Altman Clinical and Translational Research Institute, School of Medicine, University of California, San Diego, CA, United States; ^3^ Herbert Wertheim School of Public Health and Human Longevity Science, University of California, San Diego, CA, United States; ^4^ Family Health Centers of San Diego, San Diego, CA, United States; ^5^ Consulting Solutions, LLC, San Diego, CA, United States; ^6^ American Academy of Pediatrics, Chicago, IL, United States

**Keywords:** core functions and forms, EPIS framework, fidelity, program adaptation, family protective factors, federally qualified health care centers

## Abstract

There are numerous frameworks for implementing evidence-based practices (EBPs) in novel settings to achieve “fidelity.” However, identifying appropriate referents for fidelity poses a challenge. The Core Functions and Forms paradigm offers a model that can inform adaptation decisions throughout all phases of the Exploration, Preparation, Implementation, Sustainment (EPIS) framework. We applied the Core Functions-Forms paradigm throughout the Exploration and Preparation phases of EPIS in the design of two EBPs targeting family protective factors among Latinos in San Diego, as well as describe plans for its use in Implementation and Sustainment. We employed a distinct approach for each intervention element to contrast adaptation decisions that prioritize adherence to either form or function fidelity. We describe our application of the functions-forms paradigm within the EPIS framework, focusing on the Preparation phase. We also provide functions-forms matrices that map out the relationship between individual intervention components (forms) and the essential processes (functions) by which components are theorized to exert their impact. This case study of how the core functions-forms framework can be mapped onto EPIS can support a conceptual shift from prioritizing form fidelity to also focusing on function fidelity. This might allow interventionists to target appropriate fidelity referents when adapting an EBP, rather than defaulting to maintaining fidelity to forms as described in the protocol. We see great promise for using this framework for guiding actions throughout all EPIS phases and informing future applications of this paradigm to foster more robust fidelity to function.

## Introduction

### Conceptualizing the elements of evidence-based practices

Reproducing, implementing, and sustaining the effectiveness of evidence-based practices (EBPs) within real-world contexts poses significant problems (1, 2). Although interventions are generally first developed and tested in optimal settings via efficacy trials, the contexts in which implementation ultimately occurs often deviate from this ideal. This produces concerns that intervention efficacy will be compromised through translation into real-world settings (3). These concerns have increasingly spurred interventionists to consider how research findings might be translated into clinical practice more effectively (4).

These considerations often frame the implementation and sustainment of EBP impact in novel settings as a balancing act: keeping the intervention the same by prioritizing fidelity to the protocol, on one hand, while allowing for change through adaptations to local context, on the other. This framing distinguishes between the core components of a program that are theorized to be responsible for its effectiveness, and the adaptable elements that are theorized to be modifiable without compromising impact (5–8).

This attention to core vs. adaptable elements has sparked the development of frameworks that provide guidance on distinguishing between core and adaptable intervention elements [Components & Rationales for Effectiveness (CORE) Fidelity Method; Center for Substance Abuse Prevention's (CSAP) guidelines; Assessment, Decision, Adaptation, Production, Testing (ADAPT); Map of Adaptation Process (MAP)] (9–14). However, EBPs are rarely evaluated in such a way that allows for retroactive disentanglement of these elements, particularly in a way that allows for a clear approach to define which elements are truly “core” vs. “adaptable” (15). This leaves implementers without sufficient guidance around what must be maintained and what can be adapted, often defaulting to prioritizing fidelity to the prior protocol while giving little attention to adaptation for fit with patient population or service context (7, 16). While there is value in establishing fidelity referents based on prior operationalizations and having EBP developers identify what they view as core vs. adaptable, there are potential unintended consequences.

Most critically, determination of what is “core” vs. “adaptable” is, in itself, likely context-dependent. This means that, while there is value in an original intervention developer specifying their hypotheses on what is “core” vs. “adaptable”, their assumptions are likely influenced by the context of the original intervention's setting and patient population; while it is plausible that what is core in that context will be core in another context, it is, by no means, definitionally true. Basing intervention fidelity in novel settings on prior operationalizations might bias interventionists towards what was appropriate in “ideal” conditions for that operationalization and not what is appropriate in the present context. Thus, there is a risk that robust locally adapted EBPs that are better aligned with a particular context will not be attempted due to a commitment to overly rigid fidelity to the original operationalization of a given EBP.

To address this issue, we argue for an alternative approach to help guide adaptation of EBPs to local contexts; namely the theorized mechanism of action or function of any given intervention element as an alternative fidelity referent. Monitoring the desired purpose or function of an intervention element opens a broader array of possible operationalizations of forms that could achieve the intent of the core function. This approach then provides a pathway that both allows for more localized adaptation of intervention elements (i.e., to create new forms) while still guiding fidelity to hypothesized functions.

### The Core Functions and Forms paradigm

The “Core Functions and Forms” model offers one such approach to establishing an alternative fidelity referent. It represents a paradigm shift in how we define interventions by distinguishing between their core functions (i.e., the underlying elements of an EBP hypothesized to be responsible for achieving its proximal, theorized mechanism of action) and their associated forms (i.e., the specific operationalization/protocol of intervention elements intended to enact the core function) (17). This model has primarily been applied by either retroactively identifying functions and forms to generate function-form matrices or prospectively guiding pre-implementation adaptations (17–19). Theorists have suggested furthering the use of the function-form model as a more comprehensive adaptation method, which can promote a shift from maintaining strict fidelity to an EBP's protocol (form fidelity) to fidelity to the underlying purpose of the intervention components (function fidelity) (7, 20).

This framing explicitly distinguishes between two distinct fidelity referents: forms and core functions. While interventionists have traditionally prioritized form fidelity by reproducing an established EBP's form based on its prior operationalization, function fidelity can be a complementary referent for guiding implementation adaptation to local contexts. This referent would prioritize maintenance of core functions (function fidelity) when an EBP is implemented in a novel setting, while allowing for variability in its forms according to the local context (i.e., the priorities, preferences, and resources of the EBP's adopters and its recipients). We sought to explore the potential value of each of these fidelity referents throughout all stages of EBP implementation.

### The EPIS implementation conceptual model

The Exploration, Preparation, Implementation Sustainment (EPIS) implementation framework offers a foundational conceptual model upon which we can overlay the function-form paradigm (21, 22). By expanding the scope of the intervention design and adaptation process beyond the pre-implementation period (i.e., EPIS Exploration and Preparation), EPIS allows for iterative assessments of interventions’ functions and forms throughout all four phases of implementation of an EBP (23). Furthermore, its attention to contextual factors, both within an implementing organization itself (inner context) and within the broader environment (outer context), provides a framework for identifying facilitators and barriers (a.k.a., determinants) to implementing and sustaining an intervention (24). These implementation determinants can directly inform the collaborative development and/or selection of appropriate forms (a.k.a., adaptations) that match a local context and population while still seeking to maintain sufficient fidelity to the interventions’ core functions to promote effective outcomes.

We hypothesized that we could augment each EPIS phase with the function-form approach in the following ways:

• Exploration: Focusing on both function and form can help guide EBP selection towards EBPs that have core functions that align with the goals of a local context while also utilizing forms that are judged by the team to be possible to adapt to the local context.
• Preparation: The function-form approach provides a structure (i.e., the matrix) for guiding decision-making, both for how to develop a localized adaptation of an EBP, and to guide the development of measurement protocols for monitoring fidelity to both form and function (16).
• Implementation: Consideration of both function and form can inform strategies for collecting, analyzing, and feeding back relevant data to implementers to identify where pre-planned and ad-hoc adaptations impact implementation, service, and clinical outcomes (16).
• Sustainment: Analyzing process and outcomes data can evaluate the respective levels of function and form fidelity. With this, insights on what appeared to truly be “core” of the EBP vs. “adaptable” of the EBP in this new context can be hypothesized and used to help guide further iterative localized implementation and help guide other implementation efforts in different contexts.

### The present study

To the best of our knowledge, integrating the function-form paradigm with the EPIS model has not yet been done. As such, our team operationalized the function-form approach within a study guided by EPIS as a first attempt and example for integrating the function-form approach within EPIS. Specifically, the present study is focused on adaptation of EBPs for strengthening family protective factors against adverse childhood experiences (ACEs) among Latino families in San Diego, California (25). Our work was embedded within the larger Healing Experiences of Adversity Among Latinos (HEALthy4You; H4Y) Study, which emerged as a partnership between researchers at University of California, San Diego (UCSD); Family Health Centers of San Diego (FHCSD), San Diego's largest federally qualified healthcare center (FQHC) system; the San Diego County Childhood Obesity Initiative (SDCOI), a multi-sector coalition addressing children's health through collective impact; the American Academy of Pediatrics (AAP); and the Comité Organizador Latino de City Heights (COLCH), with funding from the California Institute for the Advancement of Precision Medicine (CIAPM), focusing on addressing ACEs and health outcomes.

Team members from these organizations partnered to co-design an intervention protocol that built on FHCSD's foundational weight loss program, “Healthy Together” to target contributors to ACEs within San Diego's Latino community. One goal was to identify EBPs that could augment the Healthy Together program to address social determinants of health (SDoH) issues that impede program success and foster protective factors that can improve child outcomes, including childhood obesity. These protective factors are operationalized using the Parents’ Assessment of Protective Factors (PAPF), which has an overall aggregate “protective factors” scale along with several sub scales, including parental resilience; social connections; concrete support in times of need; social and emotional competence of children; and knowledge of parenting and child development (25).

The purpose of this exploratory sub-study was to conduct a case study report on how we integrated the core functions and forms approach when using EPIS within the H4Y study. Specifically, our case study provides a structured example of how the function-form paradigm could be used across the EPIS implementation framework, capturing how we’ve applied the function-form paradigm in the Exploration and Preparation phases, as well as how we intend to use it in the Implementation and Sustainment phases moving forward.

Beyond providing a structured example of how the function-form paradigm could map on to EPIS, a secondary goal of this work is to provide a process model of how integrating these two frameworks can guide implementation efforts that account for both function and form fidelity to produce positive outcomes. In the process, we outline our development of function-form matrices for each H4Y intervention arm, as well as provide recommendations based on lessons learned from our study for how others might further this application of the function-form paradigm for improving implementation of EBPs in real-world contexts.

## Methods

In this paper, we describe the process and insights from applying the function-form approach during the EPIS Exploration and Preparation phases that informed our development of function-form matrices. We also describe our proposed mixed- method approach for measuring both function fidelity and form fidelity during the Implementation phase. At the time of writing, the trial is in the Implementation phase. Thus, we focus on the formative work that can serve as a model for combining EPIS with the function-form approach and tools for application of the function-form paradigm throughout all EPIS phases.

### The Exploration phase

#### Stage 1: identify core functions of the overall intervention

Conceptualizing the H4Y project through the lens of functions and forms began during the EPIS Exploration phase at the earliest stages of intervention adaptation and design. Project partners from UCSD, FHCSD, SDCOI, AAP, and COLCH assembled to assess the underlying intent of H4Y in response to community needs as identified by our community-based partner organizations SDCOI and COLCH with the goal of assessing the core functions that the overall project should serve for Latino families who receive care at FHCSD. This process identified the foundational goal of providing participants with the knowledge, skills, and resources that would support the development of protective factors that have been shown to reduce risk for ACEs (25). These include parental resilience, social connections, concrete support in times of need, children's social and emotional competence, and knowledge of parenting and child development (26–29). As an important part of EPIS, a critical value in this process was assuring that power differentials and perspectives of different collaborators were considered, acknowledged, and acted on to promote equity (30).

#### Stage 2: gather data on inner and outer contextual factors that could inform implementation feasibility

Inner and outer contextual factors were carefully considered to demarcate the landscape in which intervention components would be implemented. This entailed accounting for both the clinical and administrative context of FHCSD, as well as the broader cultural context of San Diego's Latino community. This process was enhanced by close consultation with our community partner investigators from COLCH on intervention strategies and the overall approach.

#### Stage 3: consider and select evidence-based intervention components based on their perceived ability to meet the hypothesized required core functions needed within the local context

The team then considered potential intervention components based on their perceived ability to target different aspects of the overarching core function of advancing protective factors. These decisions were made using the structure of Formal Consensus and were guided by the literature on established EBPs, theoretical frameworks that offer hypothesized linkages between functions and forms, and the expertise of project partners working in the community (31).

While components related to community organizing and advocacy were combined with Healthy Together to form the base intervention (referred to as Healthy Together+, see Supplementary Material S1), others were designated as intervention arms that served as possible additional elements that would eventually result in a stepped care model. These consisted of a parenting training arm and supportive engagement arm with a community health worker/*promotora* (henceforth we will use the *promotora* label as our focus is on supporting Latino community members)*.* Given the complementarity of these two intervention components (arms), but also the added time commitment required for each, the overall team, including community partners, FHCSD, and UCSD, developed a 2 × 2 factorial trial to test the potential individual and synergistic benefits of each intervention component (Table 1). The conceptual model of these components and the protective factors that each component is intended to target are outlined in Supplementary Material S1.

**Table 1:** HEALthy4You 2 × 2 factorial design.

		Parenting training	
		Base	Step up
*Promotora* support	Base	Healthy Together + (see Supplementary Material S1)	Parenting training
	Step up	*Promotora* support	Parenting training + *promotora support*

The parenting training step up condition was inspired by and drew materials from the Incredible Years Program (IYP) (32–34). IYP is a group-based program that has demonstrated efficacy and effectiveness in numerous randomized control trials by improving parenting behaviors and strengthening childrens' socio-emotional regulation skills aligned with the protective factors flagged as important for the overall trial and aims (35–37). The choice of the IYP was consistent with the goals of the parent grant to address ACEs while also addressing childhood obesity and was selected through consideration of this and other EBPs to address ACEs. This intervention component was intended to serve the function of improving children's social and emotional competence by providing parents with knowledge of parenting and child development, as well as skills to provide a stable and nurturing home environment (27–29).

However, careful review of the core functions and forms of IYP during the Exploration phase, particularly its group-based structure, required length of each session, and number of sessions, revealed that implementing IYP would not be feasible at FHCSD, given family time constraints, billability, and logistics. While IYP provided a robust operationalization of forms (e.g., psychoeducation, roleplaying, at-home behavioral practice) that the team collectively hypothesized would be most aligned with the desired functions for community, local context necessitated adapting the intervention beyond the strict fidelity requirements established by IYP developers. This set up a valuable and important use-case for incorporating not only form fidelity (which if used alone, would have forced the team to abandon any use of IYP) but also function fidelity. The team hypothesized that, while the forms were adapted quite a bit from the original IYP, it would still be possible to implement an approach that would achieve some of the key functions of IYP at sufficient fidelity to improve protective factors among families. For example, transitioning from the group-based approach used in IYP to individual sessions to provide greater scheduling flexibility and to directly tie sessions to the patient's chart and the payer (child).

The second step up condition, engagement with health *promotoras*, is a community health worker model tailored specifically to the Latino community in which *promotoras* work directly with community members to increase capacity building skills by providing education and facilitating connections to community resources and social services (38, 39). With these activities, the *promotoras* would provide social, instrumental, informational, and emotional support and “being there” for the families in whatever they need along with helping the families address their SDoH needs. Specifically, this intervention component served the dual functions of establishing social connections and offering concrete/practical support in times of need. The increased confidence and competence parents would feel through the support of the *promotoras* was hypothesized to increase parental resilience (40, 41).

From a function-form fidelity perspective, there is wide variation on the ways (i.e., forms) in which *promotoras* can perform their core functions. This is by design and intent as, at its core, *promotoras* are there for and align with the changing and evolving needs and strengths of the families they seek to serve. Thus, the *promotora* model emphasizes the importance of the *promotoras* being able to adapt to the needs of each family, using whatever form would work best for each family. Based on this, the team hypothesized that, function fidelity would be more critical to *promotoras’* work than maintaining fidelity to any set of prescribed forms.

### The Preparation phase

Following identification of parenting training and *promotora* sessions as the intervention components to be tested in the 2 × 2 factorial trial, we formed an Adaptation Team to delineate the functions and forms of each intervention. The team consisted of a subset of members from the larger H4Y community/research partnership with expertise in adapting IYP-based parenting training to novel contexts; dissemination and implementation science; and designing and implementing complex interventions. The master trainer of *promotoras* for community-based work (MH) and the wellness coach, a masters level health coach at FHCSD (CJN), provided critical feedback and support during this process. We held weekly meetings over the course of four months as an iterative process of disentangling the intent of these interventions from the activities by which that intent was enacted to identify sub-functions of each component and their corresponding forms, based on prior EBP protocols relevant for each study arm.

Based on the differing level of protocolization between the parenting training and *promotora* sessions, we adopted different processes for developing each of their associated matrices. While the parenting training was based on a highly protocolized EBP (i.e., IYP) with rigorous guidelines for high-fidelity implementation to the protocol, *promotoras’* responsibilities in community health settings have been much more variable and contextually responsive, which manifests in the form of less protocolized activities (42–46). Given this difference, parenting training fidelity development was guided by previously defined forms and functions as outlined in the original manual. In contrast, our *promotora* matrix started with an emphasis on fidelity to function, followed by an open discussion of possible forms that might be used to enact a given function. In discussion, either form or function was used as the starting referent and we iteratively developed a matrix that articulated both functions and forms. The following outline of the stages of the Preparation phase highlights the parallel processes of developing matrices according to each fidelity referent (function and form).

#### Stage 4: identify core functions and possible forms of each intervention component

Determinations of the inclusion and exclusion of any given function with corresponding possible forms for each function were made using a Formal Consensus approach and operationalized into a set of matrices that were iteratively developed and refined (31). This differentiation between functions and forms was particularly important as maintenance of functions is theorized to be essential to intervention fidelity, whereas the selection of the “right” form to achieve a desired function is seen as the central pathway for achieving local adaptation. Guided in part by INUS condition causality as outlined by Mackie (1965), the key goal in relation to identifying functions was to create a comprehensive list of *necessary* and *sufficient* functions that we theorized would need to be delivered to improve protective factors in relation to ACEs and childhood obesity (47). In contrast, the goal of identifying forms was to identify a broad list of observable and measurable intervention operationalizations that each alone may not be *unnecessary but would be sufficient* to enact each theorized function, thus recognizing that there are many possible forms that could be used to achieve a theorized function. For example, the function of “building parents’ capacity to serve as a social, emotional, and academic coach for their child” could potentially be addressed through any possible combination of psychoeducation, collaborative problem-solving, setting SMART goals, and/or roleplaying, depending on the context.

Using these distinctions between functions and forms, we identified core functions for each EBP. In the case of the parenting training, we started with a template of forms from the IYP lesson plan/protocol and functions based on the explicit purpose of each individual session of IYP as outlined in the manual (i.e., Serving as a Social and Emotional Coach for Your Child) (46).

In contrast, we began with identifying core functions of the *promotora* sessions by first recognizing the overarching function of *promotoras* as, “being there for the family.” From this, we iteratively identified and refined sub-functions that contribute to that high level function such as providing social, emotional, and practical (instrumental and informational) support. Concurrently, the team drew on first-hand knowledge and experience of our expert *promotora* trainer along with existing resources and training materials for *promotoras* to identify a list of possible forms that could be used to achieve each sub-function and the overall function of “being there for the family” (39).

#### Stage 5: select contextually relevant forms to fulfill the core functions of each intervention component

Using data on inner and outer contextual factors collected during the Exploration phase, we next evaluated implementability of possible forms within the FHCSD context. This involved accounting not only for the feasibility of delivering intervention sub-elements (i.e., forms) themselves, but also how we might effectively measure the extent of implementation of a form and, ideally, seek to identify indicators that the form did truly enact the targeted function in practice. Our team actively triangulated between patient, provider, institutional, and research concerns that had been identified during the Exploration phase to ensure that the interventions that could be implemented with sufficient fidelity to function as our overall focus, while recognizing that forms are what tends to be observable and measurable. While IYP offered a clearly delineated list of prescribed forms for parenting training that we could adapt for our local context, identifying appropriate forms for the *promotora* sessions primarily relied on our team member's direct experience of training and supervising *promotoras* across San Diego for over a decade and published literature on *promotora* activities. See Supplementary Material S2 for an example of a preliminary menu of relevant functions and forms developed as an intermediate product at this stage.

#### Stage 6: map out the relationship between each intervention components’ functions and forms in a matrix

Having arrived at a plausible menu of functions and forms for each intervention component, we sought to map out the relationships between them in a matrix. This entailed assigning possible forms to each function, as well as providing clarifying information regarding how the form might be enacted (and thus observed/measured) to fulfill the corresponding function.

For the parenting training, we generated hypothesized linkages between core functions and their associated menu of forms using IYP's theoretical framework outlined in the literature, namely Patterson's social learning model, Bandura's modeling and self-efficacy theories, and Bowlby's attachment theory (34, 48). We iteratively prioritized the core functions within the original IYP program that were hypothesized to be necessary to focus on for Latino families receiving care at FHCSD, based on the real-world knowledge of mental health professionals who are, at the time of writing, delivering the parenting training. From this, we engaged in continual culling and adaptation of forms that would each be sufficient to fulfill the underlying function until a final program that the mental health providers at FHCSD felt confident they could deliver within the real-world constraints of FHCSD and aligned with training options that the families they serve were hypothesized to appreciate receiving. This provided an indicator of form fidelity, which could document fidelity to the original EBP (i.e., IYP) operationalizations, as well as form adaptations.

Conversely, we proposed theoretical linkages between *promotora* functions and forms using a modified version of the 5 A's Intervention Model, which has previously demonstrated efficacy and effectiveness in providing concrete support to aid smoking cessation, as the basis of our *promotora* matrix (49–51). Although *promotoras* also offer concrete forms of support, we hypothesized that the primary function was relational. Thus, the core function a *promotora* serves is, by definition, highly context-dependent, dynamic, and potentially idiosyncratic between a *promotora* and each family. Given this overarching, highly dynamic function of “being there for the family”, we sought a theoretical framework that could be used to make this more concrete while still honoring the inherently dynamic nature of the function. This led us to the 5 A's of Ask, Advise, Assess, Assist, and Arrange as key possible sub-functions for creating relational support while allowing for continuous adaptations to their approach based on feedback from the family. Furthermore, we added three additional A's to, respectively, capture the *promotora's* ability to connect with the family (Attune); their own capacity to care for themselves during and following difficult encounters (Actualize); and create opportunities to highlight family strengths and positive experiences (Asset Identification). Critically, returning to our INUS guidance, we conceived of “being there for the family by providing social, instrumental, informational, and emotional support” as the only truly necessary and sufficient function that needed to be met in each session, which could then be achieved by any possible mix of the 8 A's as functions that could manifest via a wide variety of possible forms.

For example, the Attune function addresses the *promotora's* ability to establish a robust therapeutic alliance and relationship with the family to connect with their prioritized needs. We viewed this as key indicator of “being there for the family”, with the *promotora* serving as a trusted confidant who could provide the family with both informal emotional support through a meaningful interpersonal connection and formal instrumental support by, for example, bridging them with existing services that might otherwise be difficult to access. Or, put differently, we hypothesized that a core function of *promotoras* is to provide a therapeutic relationship outside of the context of therapy. We hypothesized that there could be many possible forms by which a *promotora* succeeds in establishing this state of attunement with the family, with each one alone being unnecessary to always be present, but potentially sufficient for achieving the core function of Attunement. These forms could range from affective stances (“engage in a non-judgmental, supportive manner,” “used compassion for self and others to support the family,”) to specific communication techniques (“use motivational interviewing techniques,” “Engage in active/intentional listening”). We hypothesized that any given combination of these forms could contribute to fulfilling the function of Attune, with *promorotoras’* selection guided by their understanding of the family's needs in any given session, as well as by their perception of their relationship with the family at that time and the perceived appropriateness of each form in that context.

Upon completion of the preliminary matrices, we shared and discussed the proposed functions and forms with the mental health providers’ supervisors and *promotoras* who would be delivering the interventions. These conversations were intended to support collaborative co-design that would increase the likelihood of feasibility, acceptability, appropriateness, direct immediate perceived benefit of their use by the providers (i.e., we sought to make things easier for providers explicitly rather than add “just one more thing” for them to do), and usability of the forms, as well as alignment between the identified core functions and the practitioners’ own conceptualization of the purpose of their role.

#### Stage 7: develop assessment tools to assess adherence to both function and form fidelity throughout implementation

Finally, we developed a battery of data collection instruments that would allow for assessing function and form fidelity throughout the Implementation phase. These assessments were based on the perspectives of three potentially relevant parties in each encounter: the family, the provider, and an outside observer, either a clinical supervisor or a research assistant. Our intention was to develop intervention and measurement forms that would be feasible, acceptable, appropriate, and usable by all involved parties, as well as allow for operationalization, observation, and assessment by the research team. This entailed adopting a pragmatic approach to fidelity measurement by seeking to develop tools that not only would foster gathering insights about form and function fidelity but also be useful for those gathering the data (52, 53). For example, we sought to develop note-taking templates that could fit into provider's everyday practices and would have clinical utility for future patient encounters. We also developed an adaptation documentation tool for the Implementation and Sustainment phases. This was modeled on the periodic reflections approach, an established method for documenting implementation phenomena via guided discussions with providers delivering the intervention (54).

### The Implementation and Sustainment phases

We intend to iteratively adapt the matrices throughout Implementation based on preliminary outcomes data and process data gathered from the fidelity monitoring measures and the periodic reflections. While these ostensibly constitute Stages 8–10, these processes will likely be co-occurring, as opposed to sequential. The qualitative data will be particularly valuable for its potential to (1) provide information about the occurrence of dynamic adaptations to intervention forms to assess which forms are enacted in practice, and (2) reveal provider perceptions regarding the impact of each form and how it might relate to the function with which it's hypothesized to be associated. This feedback can inform reevaluation of the matrices by expanding the menu of forms associated with each function and to reassess which functions are truly necessary.

Empirical data gathered throughout implementation can also validate, refute, or augment our micro-level theories on the linkages between functions and forms (55). We’ll adopt a similar process in the Sustainment phase once all final outcomes and process data is collected (Stage 11), along with data from FHCSD's leadership regarding their intentions for continuing to offer this programming, as well as the resources they’re able to allocate to these efforts (Stage 12). Collectively this data will guide amendments to the matrices, either by revising existing functions/forms or by including novel functions/forms that are specific to program maintenance (Stage 13). The key goal in the Sustainment phase, regarding function-form, will be to use the evidence to develop a refined theory and model that could be used by others for monitoring fidelity and guiding adaptation for our two targeted EBPs.

## Results

### Core functions and forms throughout EPIS

This process resulted in a framework that integrates the core functions and forms paradigm into the EPIS implementation model (Table 2). This framework demonstrates how conceptualizing an intervention according to its functions and forms can inform each phase of the implementation process. This approach plays out as an iterative process in which partners identify functions and forms with increasing granularity, starting with the overall purpose of the project in response to community needs and ultimately arriving at a series of more discrete functions and associated forms that collectively satisfy these goals in the implementation context.

**Table 2:** The core functions and forms framework throughout the EPIS phases.

	Exploration	Preparation	Implementation	Sustainment
Goal	– Achieve an understanding of what is feasible, acceptable, appropriate, usable, and theorized to be beneficial in the present context. – Select intervention components that can plausibly fulfill the project's core functions.	– Creation of preliminary function-form matrices for each intervention component. – Creation of fidelity monitoring and adaptation documentation tools to be used throughout Implementation and Sustainment that incorporates monitoring of fidelity to both form and function.	– Produce an amended matrix that reflects lessons learned throughout the process of implementation with a refined understanding of what sufficient function fidelity and form fidelity likely consist of within the specific context.	– Study the degree to which theorized fidelity to function or fidelity to form influenced EBP effectiveness on target outcomes. – Update the matrix to be used in as the foundation for fostering sufficient sustainment, the next iteration of EPIS in the local context, and as a starting point for guiding implementation of these EBPs in other contexts.
Process	Stage 1: Identify core functions of the overall intervention. Stage 2: Systematically gather information on inner and outer contextual factors in collaboration with partners. Stage 3: Consider existing evidence-based intervention components that could serve as forms through which the project's functions are fulfilled.	Stage 4: Identify core functions and possible forms of each intervention component. Stage 5: Select contextually relevant forms that correspond to the core functions of each intervention component. Stage 6: Map out the relationship between functions and forms in a matrix. Step 7: Develop assessment tools that evaluate function and form fidelity of intervention implementation	Stage 8: Gather data about fidelity to form and function and preliminary outcomes data. Stage 9: Track implementation processes and adaptations using periodic reflections. Stage 10: Iteratively adjust the function-form matrices based on outcome and process data.	Stage 11: Gather final outcomes and process data about fidelity to function and form and analyze the data with regard to both fidelity referents. Stage 12: Assess partner's intentions for sustaining the intervention. Stage 13: Further amend the matrices based on this information.

Each factor in this table is primarily within the EPIS inner context of FHCSD. However, the funding agency (CIAPM) is an important determinant from the EPIS outer context while the grant funding that supports this project can be considered as a “bridging factor” that links outer and inner context.

### Function-form matrices

We also developed matrices of core functions and associated evidence-informed forms for each intervention arm. These matrices consist of four columns, delineating (1) the core functions that each arm is hypothesized to serve, (2) the forms individual practitioners can use, (3) the assessment tools to be completed by participating families, and (4) the assessment tools to be completed by providers delivering the intervention or by their observing supervisors. Table 3 offers an abbreviated matrix that highlights key information included in each column, while the comprehensive matrices for the parenting training and *promotora* sessions are available as Supplementary Materials S3 and S4, respectively.

**Table 3:** Abbreviated matrix outlining the functions, forms, and corresponding assessment tools for evaluating *promotora* sessions.

Function	Form	Promotora assessment	Patient assessment
Attune: establish a supportive and collaborative therapeutic relationship with the family to connect with their prioritized needs	Use motivational interviewing techniques (i.e. open-ended questions, reflective statements, summarizing)	STAR-C	STAR-P
	Share personal experiences that are connected to those of the family	Promotora post-visit assessment form	** **
	Engage in active/intentional listening	** **	** **
	Follow the family's priorities throughout the session	** **	** **
Actualize: engage in self-care to bolster one's own resources when engaging with other's suffering	Write end-of-day reflection	End-of-day reflection form	** **
	Participate in promotora supervision session	** **	** **
Asset identification: create opportunities to highlight positive experiences, as well as individual, familial, and communal features that promote well-being	Identify and label family's assets and strengths	Promotora post-visit assessment form	** **
	Ask about relationships that the family finds to be meaningful and supportive	** **	** **
	Ask about forms of civic or social engagement in which the family participates and finds to be meaningful	** **	** **

## Discussion

### Advancing function fidelity throughout EPIS

The core contribution of this work is to propose a series of steps through which the function-form paradigm can be used across all phases of EPIS. While others have begun exploring the role of the function-form paradigm within the Preparation phase, addressing its role throughout EPIS offers a more comprehensive approach to advancing fidelity that can account for both form fidelity and function fidelity (7, 20). By outlining the process by which researchers can adopt the function-form perspective in each phase of EPIS while also invoking EPIS determinants (i.e., outer context, inner context, bridging factors, interconnections, linkages, and relationships of entities and people involved), the present study seeks to expand the ways in which fidelity is studied within implementation science efforts.

The key innovation in this approach is to shift fidelity monitoring away from a strong focus on fidelity to prior operationalizations of an EBP (form fidelity) to, instead, incorporating both function fidelity and form fidelity in a principled way. Specifically, we propose a focus on intervention elements’ underlying functions as the core referent (function fidelity), which, together, are theorized to be a *necessary and sufficient* set to produce the desired effects of an intervention package. This focus is complemented by developing a list of forms, which are sufficient for enacting a given function but not strictly necessary. This list offers an approach to fidelity monitoring that enables documentation of *a priori* and *ad hoc* adaptations, with justifications for said changes guided by fidelity to function, as well as feasibility in the local context. With this, function becomes the hypothesized core, while the forms are treated as adaptable to local contexts.

Contrasting our development processes of each matrix also highlights how either form fidelity or function fidelity can inform adaptation decisions. Our development of the parenting training matrix started with a greater emphasis on form fidelity, in line with the method employed by other users of the function-form approach. This consists of deconstructing complex interventions into their constituent forms for discrete assessment, followed by assessment of which underlying function each form is intended to fulfill (17, 19, 56, 57). For example, Kirk and colleague's (2021) case study of applying the function-form framework to streamlining the process of hospice referrals was guided by a literature review that identified forms listed in candidate intervention protocols, followed by qualitative interviews that could inform the *post hoc* identification of underlying functions (19). This form fidelity approach parallels other advancing methods for disentangling the building blocks of interventions so that they can be reassembled for best fit with the implementation environment, such as the common elements approach (58).

In contrast, our development of the *promotora* matrix represents a reversal of this approach. We began with identifying the overall hypothesized function of “being there for the family”, followed by a literature review to select a conceptual framework for understanding *promotora* interactions (5 A's) as key sub-functions. Finally, we then drew on the literature and our team's firsthand expertise as master *promotoras* and *promotora* trainers to develop a list of plausible forms of the types of interactions and services a *promotora* may offer within each of the sub-functions. This inverted approach was guided by the belief that transferring effectiveness of interventions across contexts primarily hinges on retaining their core functions, rather than the forms by which they manifest, coupled also with the highly context-dependent, dynamic, and feasibly idiosyncratic nature of the core function of “being there for the family.”

The dynamic nature of the core function of *promotoras* set up an important need to start with function fidelity and to then devise an approach to form fidelity that could honor and match the inherent complexity of the hypothesized function. While prioritizing fidelity to function over form and vice versa can, respectively, guide adaptation and monitoring efforts, maintaining consideration of both fidelity referents throughout all EPIS stages supports collaborative co-design with project partners. This dual focus on the goal of the EBP and how it will be pragmatically implemented and monitored creates a space for actively and continuously involving those who are directly involved in either delivering or receiving the intervention, which has been an area of increasing focus within the implementation science literature (4, 59). Although our study focused on Latinos in San Diego, this approach could be similarly adapted to various ethnic groups and populations to co-design interventions that are relevant and effective for meeting their unique needs. Moreover, our structure of developing and iteratively modifying the matrices in partnership with the individuals who would be directly involved in its delivery fostered a shared respect for the project's goals and priorities. This approach also contributed to the creation of research materials that could be feasibly incorporated into the workflow of the providers who would ultimately be putting them into practice. This was especially important given our intent to center the priorities and values of our clinical partners over our research goals to promote pragmatic implementation and sustainment.

### Recommendations for furthering the function-form paradigm as a method

This study was primarily exploratory in integrating the function-form model with the EPIS framework. While focused on its application to EPIS, the function-form framework has significant overlap with other implementation science models and there are many possibilities for synthesizing these approaches to promote a method that recognizes the dynamic relationship between functions and forms while simultaneously maintaining a high degree of methodological rigor. Furthermore, in the absence of an existing theoretical framework to guide selective emphasis of form or function fidelity, we offer the following recommendations on when each referent might take greater precedence across each EPIS phase and the ways in which our approach could be strengthened through augmentation with other implementation sciences methods.

During Exploration and Preparation, we propose that prioritizing function fidelity is essential to carefully identifying the key hypothesized drivers of change that are feasible to be implemented in a local context. Although we were limited in our ability to review, evaluate, and select intervention components based on existing evidence in the literature, we would recommend that future adopters of this approach follow more rigorous guidelines for assessing candidate forms to establish evidence-informed inclusion criteria (19, 58). Edmunds’ et al.'s (2022) Components & Rationales for Effectiveness (CORE) Fidelity Method for identifying key components of EBPs offers highly relevant guidance for augmenting our approach by providing systematic guidelines for gathering and synthesizing information to develop a CORE model that outlines the essential intervention components to fulfilling each function and the possible forms by which this might occur (14).

Although the CORE Method was not published until our project was already underway, which precluded incorporation of its guidelines into our own method, there are fruitful possibilities for integrating our approaches. This integration could capitalize on the CORE Method's rigor, including its systematic approach to literature review and component selection using the Nominal Group Technique, while simultaneously benefiting from our method's expanded scope beyond EPIS’ Preparation phase and our active inclusion of community partners throughout intervention development (14, 60).

Moving into Implementation, function fidelity remains central to retaining the “secret sauce” as the EBP's forms undergo *ad hoc* adaptations to meet the local context. Specifically, while function fidelity is the focus, on-going examination of the degree to which various forms are included and whether they’re adapted can likely provide valuable insights on how to engage in principled adaptations throughout Implementation. These efforts would be supported by adopting a more refined assessment approach that incorporates recent advancements in implementation science that provide a framework for operationalizing adaptations [e.g., Adaptome (61); Model for Adaptation Design and Impact (MADI) (62); Framework for Adaptation and Modifications (FRAME) (63, 64); Dynamic Adaptation Process (DAP) (16)]. Such a systematic approach would promote greater confidence in linking functions with the forms that are ultimately delivered. It would also promote more accurate disentanglement of functions from forms.

During the Sustainment phase, we suggest the use of INUS condition logic to guide analyses to study *a priori* hypotheses and generate new hypotheses regarding function, form, and feasibility (47). In terms of a testing *a priori* hypotheses, the function-form matrix presents a range of hypotheses to analyze: if function fidelity is predictive of improved outcomes; if form fidelity is predictive of the corresponding function fidelity; and the key forms and functions that independently or synergistically produced desired effects, using secondary analysis techniques [e.g., those used in prior meta-analyses on the subject (14, 65, 66)]. This process of testing and refining *a priori* hypotheses can be informed not only by data collected throughout Implementation and Sustainment, but also by using existing mid- and macro-level theories to further contextualize function-form linkages within a broader evidence base. Doing so would increase confidence in the validity of the micro-theories explaining how forms fulfill functions or serve as an impetus to modify these theories to bring them in closer alignment with established models.

Conducting exploratory analyses during Sustainment can also generate new hypotheses that outline evidence-informed formulations of the true functions of an EBP and the conditions under which any given form should be implemented to fulfill those functions. Within these exploratory analyses, the goal would be to identify functions that are independently necessary and, together, are sufficient to produce desired effect. Further, exploratory analyses can study if forms were *feasible to enact in a local context* and *sufficient to produce their targeted function*. This allows for developing more thoughtful formulations on how to adapt the EBP that could inform subsequent work both in the local context and, eventually, facilitate more robust conceptual models to guide adaptation efforts.

We recognize that this approach closely parallels Linda Collins’ multiphase optimization strategy (MOST) and its innovative use of optimization trials, such as factorial trials, sequential multiple assignment randomized trials (SMART), micro-randomized trials, and system identification experiments (67). These optimization trials are defined by the use of a clearly specified and testable optimization criterion(ia). For example, in the classic MOST experiment of using a factorial trial as a screening experiment, interventionists establish the optimization criterion that defines an optimized intervention package as one that includes only intervention components that have demonstrated the ability to produce desired effects (i.e., the “no dead weight” criterion). The “no dead weight” criterion is directly analogous to our “necessary and sufficient” criterion we propose for assessing functions. Thus, one logical extension to this work could be to use results gleaned from the analyses described above to guide the development of screening experiments that rigorously test which functions truly are necessary and sufficient to produce desired effects. Hypothesized core functions would then serve as optimization criteria that can then be used as benchmarks when evaluating local adaptations with the use of the “no dead weight” optimization criterion as a logical way of operationalizing such a test. This is all purely speculative at present and would benefit from further work that explores the use of function fidelity as an optimization criterion.

In closing, we recognize that the rigor involved in adopting such a systematic approach to screening and monitoring the function and form fidelity of intervention elements may preclude its use in projects that cannot dedicate sufficient resources to studying implementation. As such, developing more pragmatic methods of fidelity monitoring and adaptation tracking represents an important next step for improving the accessibility of these practices beyond the realm of implementation science and enhancing the feasibility of this approach. This is another potential area of promising integration between our approach and MOST, which adheres to the “resource management principle” that dictates that researchers must always seek to make the best and most efficient use of all available resources in their investigations (68). This translates into a prioritization of pragmatism in which the level of investigative rigor corresponds to the rigor needed to support evidence-based decision-making. Within this framing, a full screening experiment may not be justifiable and other more pragmatic approaches, such as what we have proposed, would be appropriate to guide localized EBP adaptation, implementation, and fidelity monitoring decisions. This could lower barriers to rigorously adapting, optimizing, and monitoring EBPs in novel contexts across all phases of EPIS, which would not only deepen EBP impact, but also further promote the involvement of community partners who have been historically less involved in implementation science research initiatives.

## Conclusion

Implementing EBPs in novel settings inherently presents challenges regarding the degree to which an intervention can be adapted while producing targeted effects. The core functions and forms paradigm can help to alleviate these challenges, serving not only as a framework for retroactively disentangling essential and adaptable elements, but also for establishing a novel fidelity referent, function, that can inform adaptation decisions throughout the four phases of EPIS (Exploration, Preparation, Implementation, Sustainment). This case study operationalized the function-form paradigm as an implementation method within the context of two interventions targeting family protective factors among Latino children throughout the Exploration and Preparation phases, while also reflecting on its applications throughout Implementation and Sustainment.

Our development of matrices that outline the core functions and associated forms of each EBP guided adaptation and implementation decisions, spurring the recommendation to attend to both function and form fidelity throughout EPIS. This appreciation for dual fidelity referents would be further supported by integrating aspects of the CORE Method and MOST to promote more rigorous screening and selection of intervention components for inclusion and more pragmatic optimization and evaluation of EBPs in novel contexts. We hypothesize that these steps can enhance the applications of this novel approach to overcoming the difficulties of balancing evidence of effectiveness with contextually-necessary adaptations.

## Data Availability

The original contributions presented in the study are included in the article/Supplementary Material, further inquiries can be directed to the corresponding author.
