# Supplementary figures and images for: The core functions and forms paradigm throughout EPIS: designing and implementing an evidence-based practice with function fidelity
Source: Front Health Serv. 2024 Jan 16;3:1281690. doi: 10.3389/frhs.2023.1281690 (PMC10826509; doi:10.3389/frhs.2023.1281690)

## Appendix 1: HEALthy4You Intervention Components and Corresponding Protective Factor Targets

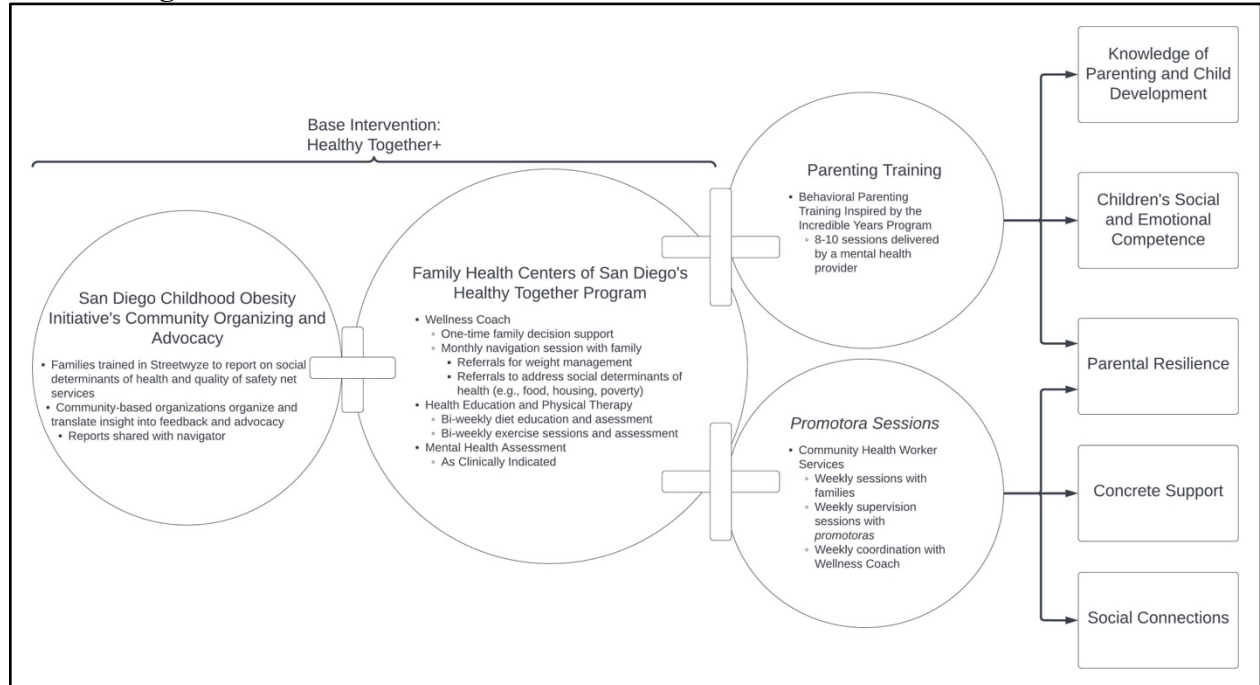

Supplement: Supplementary file 1 [file Datasheet1.pdf]
