# Supplementary material for: The core functions and forms paradigm throughout EPIS: designing and implementing an evidence-based practice with function fidelity
Source: Front Health Serv. 2024 Jan 16;3:1281690. doi: 10.3389/frhs.2023.1281690 (PMC10826509; doi:10.3389/frhs.2023.1281690)
Supplement: Supplementary file 2 [file Datasheet2.pdf]

Appendix 2: Preliminary Menu of the Functions and Forms of Relevance to *Promotora* Sessions

Function	Form
Attune to parental needs and challenges	Open-ended questions
Offered to provide instrumental support aligned with family's priorities	Active listening
Connect emotionally with parent	Reflective statements
Provide emotional support	Summarizing
Build parent's capacity for emotion and stress regulation	Provide information
Offered to broaden/strengthen parent's social and resource network	Provide referrals
Build parent's self-efficacy to manage life stressors	Assist parents in breaking down barriers
	Grounding interactions in personal/lived experience
	Empathic listening skills
	Identify assets and strengths
	Use of grounding exercises/practices
