# Supplementary material for: The core functions and forms paradigm throughout EPIS: designing and implementing an evidence-based practice with function fidelity
Source: Front Health Serv. 2024 Jan 16;3:1281690. doi: 10.3389/frhs.2023.1281690 (PMC10826509; doi:10.3389/frhs.2023.1281690)
Supplement: Supplementary file 3 [file Datasheet3.pdf]

Appendix 3: Parenting Training Matrix Outlining the Functions, Forms, and Assessment Tools from Provider and Patient Perspectives

Function	Form	Provider Assessment	Patient Assessment
To build positive rapport with both parent and child	Psychoeducation<br>Problem-Solving<br>Behavioral Practice Using Worksheets/Handouts	STAR-C<br>All EMR Notes<br>Periodic Reflections	STAR-P<br>Reported Use of Handout/Exercise:<br>Positive Self-Statements Brainstorm<br>Incentives for Me Brainstorm<br>Self-Talk in Problem Situations
To help strengthen parent-child bond/connection	Psychoeducation<br>Problem-Solving<br>Set a SMART Goal<br>Roleplay<br>Behavioral Practice Using Worksheets/Handouts	STAR-C<br>EMR Notes Addressing:<br>Parental Attention<br>Persistence Coaching<br>Effective Praise<br>Tangible Rewards<br>Periodic Reflections	STAR-P<br>PAPF<br>Reported Use of Handout/Exercise:<br>Special Time Record Sheet<br>Evaluating Special Time Interactions Checklist<br>Play and Special Time Brainstorm
To build parent capacity to serve as a social, emotional, and academic coach for child	Psychoeducation<br>Problem-Solving<br>Set a SMART Goal<br>Roleplay<br>Behavioral Practice Using Worksheets/Handouts	EMR Notes Addressing:<br>Persistence Coaching<br>Managing Your Own Emotions<br>Periodic Reflections	Reported Use of Handout/Exercise:<br>Praise Record Sheet<br>Emotional Literacy Checklist<br>Social Skills Coaching Checklist<br>Academic Coaching Checklist<br>Labeled Praise Refrigerator Notes<br>Positive Self-Statements Brainstorm
To build parent confidence and skills in providing labeled praise to build child's social, emotional, and academic competency	Psychoeducation<br>Problem-Solving<br>Set a SMART Goal<br>Roleplay<br>Behavioral Practice Using Worksheets/Handouts	EMR Notes Addressing:<br>Effective Praise<br>Managing Your Own Emotions<br>Periodic Reflections	Reported Use of Handout/Exercise:<br>Praise Record Sheet<br>Behavior Record Sheet<br>Praise Brainstorm<br>Positive Self-Statements Brainstorm<br>Providing Praise Refrigerator Notes
To build parent confidence and skills in providing incentives and rewards to build child's social, emotional, and academic competency	Psychoeducation<br>Problem-Solving<br>Set a SMART Goal<br>Roleplay<br>Behavioral Practice Using Worksheets/Handouts	EMR Notes Addressing:<br>Tangible Rewards<br>Managing Your Own Emotions<br>Periodic Reflections	Reported Use of Handout/Exercise:<br>No-Cost/Spontaneous Tangible Rewards Brainstorm<br>Incentives for Me Brainstorm<br>Rewards Chart<br>Tangible Rewards Refrigerator Notes

			Behavior Record Sheet
To help parents establish household rules, routines, and responsibilities	Psychoeducation<br>Problem-Solving<br>Set a SMART Goal<br>Roleplay<br>Behavioral Practice Using Worksheets/Handouts	EMR Notes Addressing:<br>Rules and Routines<br>Periodic Reflections	Reported Use of Handout/Exercise:<br>Written morning routine for child<br>Written bedtime or after-school routine for child<br>Written household rules of family
To help parents set clear limits for their child in their home	Psychoeducation<br>Problem-Solving<br>Set a SMART Goal<br>Roleplay<br>Behavioral Practice Using Worksheets/Handouts	EMR Notes Addressing:<br>Clear Limit Setting<br>Managing Your Own Emotions<br>Periodic Reflections	Reported Use of Handout/Exercise:<br>Limit Setting Brainstorm<br>Effective Commands Brainstorm<br>Limit Setting Refrigerator Notes<br>Commands Record Sheet
To build parent capacity to handle misbehavior and use discipline appropriately	Psychoeducation<br>Problem-Solving<br>Set a SMART Goal<br>Roleplay<br>Cognitive-Emotional Regulation Training<br>Behavioral Practice Using Worksheets/Handouts	EMR Notes Addressing:<br>Logical Consequences<br>Periodic Reflections	Reported Use of Handout/Exercise:<br>Ignoring Misbehavior Brainstorm<br>Staying Calm While Ignoring Brainstorm<br>Selective Attention Brainstorm<br>Ignoring Misbehavior Refrigerator Notes<br>Behavior Record<br>Self-Talk in Problem Situations Handout<br>Ignore and Praise Record Sheet<br>Logical Consequences Refrigerator Notes<br>Special Time Record Sheet<br>Logical Consequences Record Sheet<br>Work Chores Record Sheet
To build parent's capacity to problem solve difficulties with their child	Psychoeducation<br>Problem-Solving<br>Set a SMART Goal<br>Roleplay<br>Behavioral Practice Using Worksheets/Handouts	EMR Notes Addressing:<br>Problem Solving<br>Managing Your Own Emotions<br>Periodic Reflections	Reported Use of Handout/Exercise:<br>SOLVE Practice Sheet
To build parent confidence and skills to manage child's emotions	Psychoeducation<br>Problem-Solving<br>Set a SMART Goal<br>Roleplay	EMR Notes Addressing:<br>Persistence Coaching<br>Managing Your Own Emotions<br>Periodic Reflections	Reported Use of Handout/Exercise:<br>Emotional Literacy Checklist

	Behavioral Practice Using Worksheets/Handouts		
To build parent confidence and skills to manage their own emotions	Psychoeducation	EMR Notes Addressing: Managing Your Own Emotions	Reported Use of Handout/Exercise: Emotional Literacy Checklist
	Problem-Solving		
	Set a SMART Goal	Periodic Reflections	
	Roleplay		
To improve parent communication skills	Psychoeducation	EMR Notes Addressing: Persistence Coaching, Effective Praise, Clear Limit Setting, Managing Your Own Emotions	Reported Use of Handout/Exercise: Emotional Literacy Checklist, Social Skills Checklist, Praise Brainstorm
	Problem-Solving		
	Set a SMART Goal		
	Roleplay		
	Behavioral Practice Using Worksheets/Handouts		Positive Self-Statements Brainstorm
		Periodic Reflections	

SMART Goal: Specific, Measurable, Achievable, Relevant, and Time-Bound Goal
STAR-C: Scale to Assess Therapeutic Relationship - Clinician
STAR-P: Scale to Assess Therapeutic Relationship - Patient
EMR: Electronic Medical Record
PAPF: Parents' Assessment of Protective Factors
