# Supplementary material for: The core functions and forms paradigm throughout EPIS: designing and implementing an evidence-based practice with function fidelity
Source: Front Health Serv. 2024 Jan 16;3:1281690. doi: 10.3389/frhs.2023.1281690 (PMC10826509; doi:10.3389/frhs.2023.1281690)
Supplement: Supplementary file 4 [file Datasheet4.pdf]

Appendix 4: *Promotora* Sessions Matrix Outlining the Functions, Forms, and Assessment Tools from Provider and Patient Perspectives

Function	Form	Promotora Assessment	Patient Assessment
**Attune:** Establish a supportive and collaborative therapeutic relationship with the family to connect with their prioritized needs	Use motivational interviewing techniques (i.e. open-ended questions, reflective statements, summarizing)	STAR-C	STAR-P
	Share personal experiences that are connected to those of the family	Promotora Post-Visit Assessment Form	
	Engage in active/intentional listening		
	Follow the family's priorities throughout the session		
	Experience a sense of self-efficacy in being able to address family's stated needs		
	Identify and label family's assets and strengths		
	Used compassion for self and others to support the family		
	Engage in a non-judgmental, supportive manner		
	Engage in collaborative problem-solving with the family		
**Actualize:** Engage in self-care to bolster one's own resources when engaging with other's suffering	Write end-of-day reflection	End-of-day Reflection Form	
	Participate in promotora supervision session		
**Asset Identification:** Create opportunities to highlight positive experiences, as well as individual, familial, and communal features that promote well-being	Identify and label family's assets and strengths	Promotora Post-Visit Assessment Form	
	Ask about relationships that the family finds to be meaningful and supportive		
	Ask about forms of civic or social engagement in which the family participates and finds to be meaningful		
	Ask about specific spaces or environments in which the family feels a sense of safety and stability		
	Ask about recent experiences of personal growth and/or empowerment		
	Ask about family's overall well-being	Promotora Post-Visit Assessment Form	

**Agenda:** Determine the family's needs and priorities to establish an appropriate session agenda	Ask about family's priorities for the session		
	Ask about any pressing needs the family might have		
**Assess:** Determine the family's overall well-being in both HT+ and more broadly in navigating stressors	Ask about previously offered referrals	Promotora Post-Visit Assessment Form	
	Ask about previously set SMART goals		
	Ask about previously set action plans		
	Ask about current status of social determinants of health		
	Ask about overall experience with HT+ activities		
	Ask about challenges with suggestions from FHCSD providers		
	Ask about challenges or barriers family faces to living a happy, healthy life		
**Advise:** Provide support that aims to meet family's material, emotional, and lifestyle needs	Provide parenting support or health education on HT+ topics (i.e. diet, physical activity, mental health management)	Promotora Post-Visit Assessment Form	6-Month Survey Item in WCA
	Provide emotional support through empathetic statements		
	Provide emotional regulation strategies (i.e. mindfulness or compassion techniques)		
	Offer instrumental support through care coordination		
	Offer instrumental support through a referral		
**Assist:** Co-develop concrete approaches with the family to address identified needs and goals	Develop or refine SMART goals	Promotora Post-Visit Assessment Form	
	Discuss non-SMART goals		
	Develop or refine an action plan		
	Discuss plans more generally (i.e. not an action plan specifically)		
	Identify and label family's assets and strengths		
	Engage in collaborative problem-solving with the family		

	Identify possible challenges or barriers to meeting established goals or action plans		
	Develop back-up plans in case the family cannot meet their established goals or action plans		
**Arrange:** Facilitate the family's transition out of the session and prepare for future sessions	Schedule a follow-up session	Promotora Post-Visit Assessment Form	
	Share materials relevant to the material		
	Summarize key goals made during the session		
	Summarize key action plans made during the session		

STAR-C: Scale to Assess Therapeutic Relationship - Clinician
STAR-P: Scale to Assess Therapeutic Relationship – Patient
WCA: Well Child Assessment
FHCSD: Family Health Centers of San Diego
